# Thermal Conductivity of Saturated Liquid Toluene by Use of Anodized Tantalum Hot Wires at High Temperatures

**DOI:** 10.6028/jres.105.029

**Published:** 2000-04-01

**Authors:** R. A. Perkins, M. L. V. Ramires, C. A. Nieto de Castro

**Affiliations:** National Institute of Standards and Technology, Boulder, CO 80303, USA; Departmento de Química e Bioquímica and Centro de Ciência e Tecnologia de Materiais, Faculdade de Ciências da Universidade de Lisboa, Campo Grande Ed. C1, Piso 5, 1700 Lisboa, Portugal

**Keywords:** anodized tantalum wire, liquid, thermal conductivity, thermal radiation, toluene, transient hot-wire

## Abstract

Absolute measurements of the thermal conductivity of a distilled and dried sample of toluene near saturation are reported. The transient hot-wire technique with an anodized tantalum hot wire was used. The thermal conductivities were measured at temperatures from 300 K to 550 K at different applied power levels to assess the uncertainty with which it is possible to measure liquid thermal conductivity over wide temperature ranges with an anodized tantalum wire. The wire resistance versus temperature was monitored throughout the measurements to study the stability of the wire calibration. The relative expanded uncertainty of the resulting data at the level of 2 standard deviations (coverage factor *k* = 2) is 0.5 % up to 480 K and 1.5 % between 480 K and 550 K, and is limited by drift in the wire calibration at temperatures above 450 K. Significant thermal-radiation effects are observed at the highest temperatures. The radiation-corrected results agree well with data from transient hot-wire measurements with bare platinum hot wires as well as with data derived from thermal diffusivities obtained using light-scattering techniques.

## 1. Introduction

Saturated liquid toluene has been widely studied and is recommended by the International Union of Applied Chemistry (IUPAC) as a reference standard for thermal conductivity from 189 K to 360 K [1]. Efforts to extend this temperature range to 553 K have been recently reported by Ramires et al. [2]. The barriers to obtaining reliable high-temperature reference standards for thermal conductivity are a lack of data from multiple experimental techniques, and increased uncertainty due to the effects of thermal radiation. Both transient and steady-state measurement techniques for the determination of thermal conductivity are susceptible to errors due to thermal-radiative heat transfer at high temperatures since temperature gradients are imposed during the measurement, and fluids such as toluene absorb and emit the associated thermal radiation [3–5]. Even though thermal-radiation errors may be present in data from transient and steady-state techniques, agreement between radiation-corrected data from these two different techniques would provide evidence of the accuracy of the data. Unfortunately, the relative uncertainty (at the level of 2 standard deviations) of available steady-state thermal conductivity data that have been corrected for radiation exceeds 1 %, which is desired for the development of reference standards [2]. As a result, only data from a single transient hot-wire instrument [4] were designated as primary data during the development of the previous reference standard by Ramires et al. [2].

Recently, thermal diffusivity has been measured for saturated liquid toluene from 293 K to 523 K using light scattering [6]. These light-scattering data have a relative uncertainty of 2.5 %; and because there are no significant thermal gradients in the sample, thermal radiation errors are not present. The thermal conductivity λ can be calculated from the thermal diffusivity *a* by using

$$\lambda =a\rho C_{p},$$ (1)

where ρ is the fluid mass density and *C_p_* is the isobaric specific heat. An accurate equation of state is available for toluene [7] to calculate ρ and *C_p_*, but uncertainties in C*_p_* must be considered during this process. The light-scattering data were not available during the development of the previous reference standard of Ramires et al. [2].

The present measurements are made using the transient hot-wire technique as used previously [4,5]. The previous measurements were made with bare 12.7 μm diameter platinum wires and were corrected for thermal radiation. The present measurements were made with anodized 25 μm diameter tantalum wires. Anodized tantalum wires have the advantage of being electrically insulated from the fluid under study. This anodized coating allows measurements of electrically conducting fluids such as water. The highest temperature at which toluene has been previously studied with an anodized-tantalum hot-wire instrument is 370 K [8]. The present measurements extend the temperature range at which anodized tantalum hot wires have been used to measure thermal conductivity from 370 K to 550 K. The use of anodized-tantalum hot wires allows some evaluation of the reliability of the thermal radiative correction for absorbing media since the anodized tantalum wires have a different emissivity (that of tantalum pentoxide) than those of the previous platinum wires, and the diameter of the tantalum wire is twice that of the previous platinum wires.

## 2. Experimental

The transient hot-wire technique is widely recognized as an accurate method to measure the thermal conductivity and thermal diffusivity of fluids. The present measurements are absolute and require only knowledge of the geometry of the hot wires, the applied power, the resistance-versus-temperature characteristics of the wires, and time. The ideal working equation is based on the heat transfer from an infinitely long line source into an infinite medium. The temperature rise of the fluid at the surface of the wire, where *r* = *r*_0_, is given [9] by

$$\Delta T_{\text{ideal}}(r_{0},t)=\frac{q}{4\pi \lambda }\ln\left(\frac{4at}{{r_{0}}^{2}C}\right)=\frac{q}{4\pi \lambda }\ln(t)+\frac{q}{4\pi \lambda }\ln\left(\frac{4a}{{r_{0}}^{2}C}\right),$$ (2)

where *q* is the power divided by the length of the wire, *t* is the elapsed time, and *C* = e^γ^ = 1.781 … is the exponential of Euler’s constant. The ideal temperature rise of the wire is linear with respect to the logarithm of elapsed time, as shown in Eq. (2). The thermal conductivity is obtained from the slope, and the thermal diffusivity is obtained from the intercept using linear regression [10]. The temperature associated with a given thermal conductivity data point is given by

$$T_{r}=T_{0}+0.5(\Delta T_{\text{initial}}+\Delta T_{\text{final}}),$$ (3)

where Δ*T*_initial_ and Δ*T*_final_ are the temperature rise at the start time and the end time of the linear region selected for the regression. The thermal diffusivity is associated with the initial cell temperature *T*_0_ and is obtained from a calibrated reference-standard platinum resistance thermometer (PRT). All temperatures in this work are reported according to the 1990 International Temperature Scale (ITS 90), and all uncertainties are expanded uncertainties at the level of 2 standard deviations (coverage factor *k* = 2, 95 % level of confidence).

The experimental cell is designed to approximate this ideal model as closely as possible. There are, however, a number of corrections that account for deviations between the ideal line-source solution and the actual experimental heat transfer. The ideal temperature rise is obtained by adding a number of corrections δ*T*i to the experimental temperature rise according to

$$\Delta T_{\text{ideal}}=\Delta T_{\text{experimental}}+\sum_{i}\delta T_{i}.$$ (4)

These temperature-rise corrections are described in detail for our case of a coated wire in Refs. [3,11,12]. Our implementation of the corrections follows these references with the following exceptions. The compression work correction δ*T*_3_ and the radial convection correction δ*T*_4_ are set to zero following the recommendations of Assael et al. [13]. The thermal-radiation correction δ*T*_5_ is described for absorbing fluids by Nieto de Castro et al. [5].

The data-acquisition system used in this work has been described previously [4], and consists of a microcomputer with a 16 bit analog-to-digital converter, three digital voltmeters, a digital power supply, and a Wheatstone bridge which contains two hot wires in opposing legs of the bridge. The two hot wires have different lengths, and the Wheatstone bridge, which is initially balanced, subtracts the resistance change of the short hot wire from the resistance change of the long hot wire. Thus, if both wires are immersed in the same fluid, the bridge response behaves as for a finite length (the difference between the wire lengths) of an infinitely long wire and the end effects arising from axial conduction are eliminated. Heating voltage is applied to the wires through the Wheatstone bridge, and the bridge imbalance is measured in 250 equal time increments. The total time for the measurements may be varied from 1 s to 40 s, allowing one to verify that the data are obtained prior to the onset of convection. The computer checks the bridge balance prior to each experiment and records the temperature of the reference thermometer and the resistance of each hot wire for calibration purposes. The cell temperature is measured with an uncertainty of 1 mK by use of a current source and a standard resistor in series with the reference-standard PRT. The cell pressure is measured with a quartz pressure transducer from 0 MPa to 70 MPa with an uncertainty of 0.007 MPa.

### 2.1 Hot-Wire Cell

The hot-wire cell used in these measurements was designed for measurements on corrosive solutions at temperatures from 300 K to 550 K at pressures up to 70 MPa. The design of the pressure vessel and temperature control system is the same as for our previous high-temperature cells [4], so it will only be briefly described here. The pressure vessel and the internal components of the hot-wire cell are constructed of nickel alloy UNS-N10276, which is particularly resistant to halides and halide salts. The cell contains long and short hot wires located within long and short cavities with diameters of 9.5 mm. The total volume of the cell and supporting pressure system is relatively small, about 50 cm^3^, to facilitate measurements on scarce or hazardous materials. There are separate voltage and current leads to each end of each hot wire to eliminate the effects of lead resistance during the calibration process. All electrical connections in the cell are spot welded. In the assembly of the tantalum hot wires (25.4 μm diameter), polytetrafluoroethane-insulated nickel/chromium alloy wire (254 μm diameter) and polyimide-insulated platinum (76 μm diameter) wire were used to make electrical connections inside the pressure system. The connections were welded, then all the bare leads were coated three times with a polyimide/polytetrafluoroethane resin, and the assembly was baked at 550 K for several hours. The baking both cured the polymer resin and annealed the tantalum hot wires from their initial hard-drawn condition. The tantalum hot wires were then anodized in aqueous citric acid with up to 50 V to produce a film of tantalum pentoxide with an estimated thickness of 70 nm. Although the temperature control and pressure systems are rated to 750 K, the upper operating temperature of the present tantalum hot-wire cell assembly is limited to 550 K because of the melting point of polytetrafluoroethane used to electrically insulate the lead wires.

### 2.2 Sample Purification

The toluene sample used in these measurements was prepared from spectroscopic-grade toluene. The toluene was further purified by distillation over calcium hydride. Calcium hydride reacts irreversibly with any water in the sample to form calcium hydroxide precipitate, which remains in the distillation flask. The principal impurity is benzene, which has a lower boiling temperature, so the initial condensate is discarded. The sample used for measurement is then collected when the inlet to the condenser is stable at the boiling temperature of toluene. The purified sample was analyzed by gas chromatography and found to have less than 50 ng/g of benzene and less than 100 ng/g of water. The sample preparation procedure was the same as in our previous measurements [4] using bare platinum hot wires.

### 2.3 Wire Calibration

Platinum is preferred for use in resistance thermometry when it is properly annealed and is free from stress because its resistance is very stable for prolonged periods of time. Tantalum has not been used widely for resistance thermometry, so the stability of its resistance must be carefully examined and characterized. The situation is further complicated since the present tantalum wires have been anodized to form a protective layer of tantalum pentoxide. The electrical resistivity of tantalum from 273 K to 1273 K has been shown to increase in proportion to the concentration of oxygen in the sample [14]. Since the concentration of oxygen is not uniform through the wire’s cross section, there is a possibility that oxygen from the anodized layer might diffuse into the bulk tantalum wire and alter its resistance. Any oxygen-diffusion process would be enhanced at higher temperatures. The present system is ideally suited for characterizing the anodized tantalum wires since the wire resistance is measured for each wire, along with the temperature from the reference PRT and the pressure from the quartz pressure transducer, during the balance cycle for each measurement. The instrument also maintains a record of the time and date of each measurement to allow examination of the stability of the wire calibration. Since the present measurements were made along the saturation line of toluene, at pressures less than 3.3 MPa, there is not enough pressure range to allow characterization of the pressure dependence. The electrical resistivity of tantalum is known to decrease in proportion to the pressure on the sample [15].

**Fig. 1:**
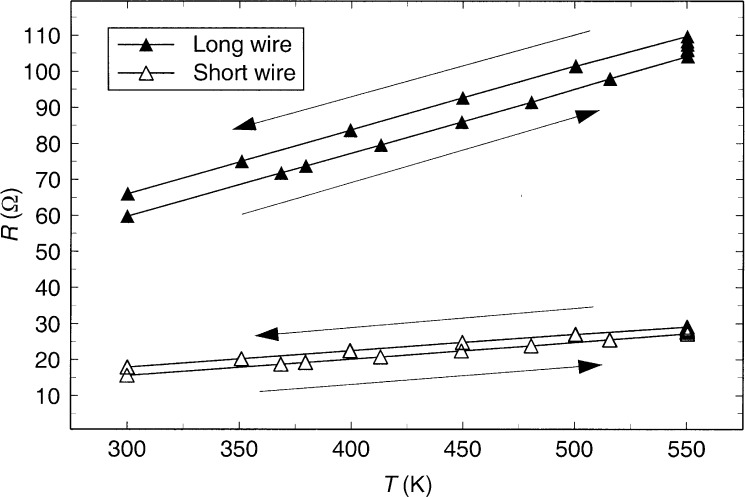
Resistance of anodized tantalum hot wires as a function of temperature. Arrows indicate the sequence of elapsed time (first increasing then decreasing temperature).

To eliminate uncertainty due to the resistance of the lead wires, there are separate current-supply and voltage-sensing leads to each end of each hot wire. The resistance is measured during the balance cycle by measuring the voltage drop across standard resistors in each leg of the Wheatstone bridge containing the hot wires, together with the voltage drop across each hot wire. The measured value is the average of five readings with a forward current of about 0.3 mA and five readings with the current reversed. This process minimizes uncertainty due to thermoelectric voltages at weld junctions and electrical connectors. The uncertainty of the resistance measurement is about 0.003 Ω. The measurements were made first with temperature increasing from 300 K to 550 K. Then, measurements were made at 550 K in the morning and evening for a period of 4 days. Finally, measurements were made with temperature decreasing from 550 K to 300 K so that hysteresis effects could be examined. The electrical resistance of the long (188.08 mm) and short (49.07 mm) hot wires are shown in Fig. 1 during this temperature cycle. It is apparent in the figure that the resistances of both the long and short hot wires increased with the elapsed time at 550 K. Although the resistance of the long wire increased more than that of the short wire, the increase was not in proportion to the wire lengths, as would be expected if the process were uniform over the entire length of each wire.

Since both the long and short hot wires come from the same sample of tantalum wire, the resistance of each wire should scale with the length of each wire. This is a requirement for use of these wires in the transient hot-wire experiment. To insure uniform heat generation over the length of each hot wire, the resistance divided by the length of the long and short hot wires respectfully must be very nearly equal. It is desirable to characterize both the uniformity of the power generation in both hot wires and the adequacy of compensation for the end effects by using two wires in different arms of the measuring bridge. This can be measured by taking the ratio σ_lw_/σ_sw_ between the resistance divided by the length of the long wire, σ_lw_ = *R*_sw_/*L*_sw_, and the resistance divided by the length of the short wire, σ_sw_ = *R*_sw_/*L*_sw_, where *R* is the wire resistance, *L* is the wire length, and the subscripts lw and sw designate the long wire and short wire, respectively. Following a previous recommendation by Kestin and Wakeham [16], σ_lw_/σ_sw_ must not deviate from unity by more than 2 %, in order to assume a correct end-effect compensation with nearly identical wires. With the definition

$$\Delta \sigma =100\left(\frac{\sigma_{\text{lw}}}{\sigma_{\text{sw}}}-1\right),$$ (5)

**Fig. 2:**
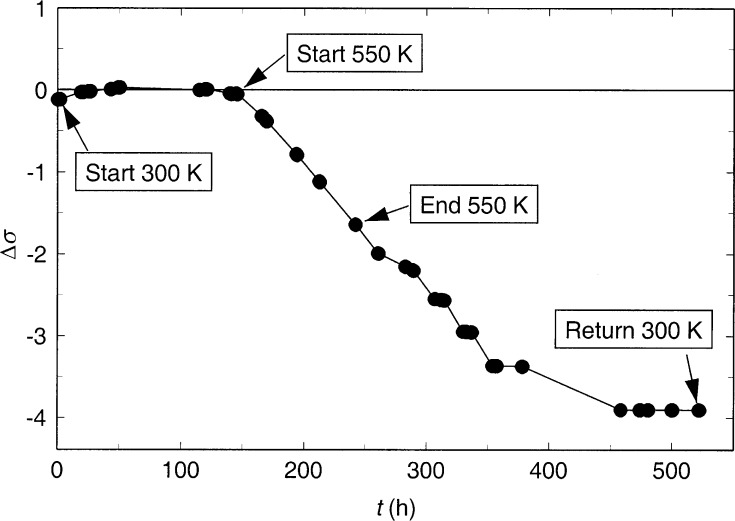
Plot of the quantity Δσ as a function of time, where Δσ = 100(σ_lw_/σ_sw_−1) with σ_lw_ = *R*_lw_/*L*_lw_ and σ_sw_ = *R*_sw_/*L*_sw_. Here *R* is the wire resistance, *L* is the wire length, and the subscripts lw and sw designate the long wire and short wire, respectively.

Figure 2 shows this percentage difference as a function of elapsed time. It can be seen that this deviation was quite stable and nearly zero during the experiments at increasing temperature from 300 K to the start of the 550 K isotherm but began to drift as the resistance of both wires increased. There was a decrease of 2 % by the end of the 550 K isotherm. This behavior is compatible with previous calculations by Kestin and Wakeham [16].

However, the increase in resistance during the 4 days at 550 K is quite dramatic and unexpected, based on our previous experience with pure platinum hot wires [4]. With pure platinum the normal behavior is a slight decrease in resistance due to annealing and stress release in the hot wires if the wires have not been at this temperature recently. Figure 2 clearly shows that the increase in resistance was not consistent with wire length. Thus, resistance increases at the welds must be considered a possibility. The only weld locations that can contribute to the measured resistance during a four terminal measurement occur where the ends of the 25 μm diameter tantalum wires are joined to the 254 μm diameter nickel-chromium alloy lead wires. Each hot wire has two welds which could potentially contribute to the measured resistance, but it is not possible to separate contributions due to the welds from those due to changes in the wires. The use of all-tantalum lead wires should be examined in the future to see whether this resistance increases within the welds or within the wires themselves.

Based on Fig. 1 and the requirement that Δσ ≤ 2 %, it was decided that only the data at increasing temperatures, including the first few hours at 550 K, should be considered for the wire-resistance calibration. The wire’s resistance was fit to a quadratic polynomial in temperature of the form *R*(*T*) = *A*_1_+*A*_2_*T*+*A*_3_*T*^2^ over four regions, 300 K to 450 K, 300 K to 480 K, 300 K to 515 K, and 300 K to 550 K. The results of these fits are given in Table 1. The resistance of a pure metal such as tantalum is known to be well approximated by such a quadratic expression [17], and the sign of *A*_3_ should be small and negative over this temperature range. Table 1 shows that the sign of the quadratic coefficients change if the resistance data above 480 K are included in the fits. This is a good indication that the increase in resistance with time becomes significant at temperatures above 480 K.

Given that the increase in resistance with time is significant at 515 K, the best possible calibration must be determined, and the influence of uncertainty in the calibration on the thermal conductivity results must be assessed. The rise in temperature at any elapsed time during a measurement is obtained from the change in resistance of the hot wires using the derivative of the calibration curve for resistance versus temperature:

$$\Delta T=\frac{\Delta R}{{\frac{\partial R}{\partial T}|}_{T}}=\frac{\Delta R}{\left(A_{2}+2A_{3}T\right)}.$$ (6)

**Fig. 3:**
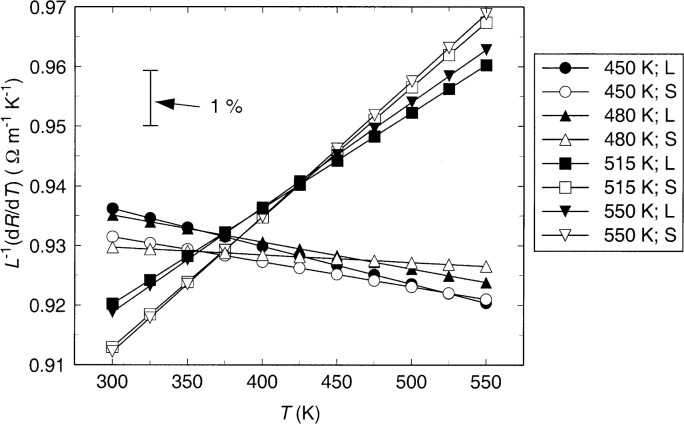
Values of $\frac{dR}{dT}$ divided by the length of anodized tantalum wire.

Thus, the uncertainty of the wire’s temperature rise, and of the measured thermal conductivity, is directly related to the uncertainty of the derivative of wire resistance with respect to temperature. This resistance derivative divided by length is plotted as a function of temperature in Fig. 3, which shows the effect of the change in sign of A_3_ when resistance data at temperatures above 480 K are included in the fit. Based on data below 480 K, where there is confidence that the calibration is stable, the resistance derivative decreases by 1 % for a temperature increase of 180 K. Since this temperature dependence is quite small, and the region of extrapolation (480 K to 550 K) is less than half this temperature range, it is anticipated that extrapolation errors should be less than 1 % if only data below 480 K are used for the calibration. If resistance data from temperatures above 480 K are used in the calibration, the thermal conductivity results at 550 K will be about 4 % higher at 550 K and results at 300 K will be 2 % too low. Based on these considerations, the wire calibration from 300 K to 480 K is used in the subsequent data analysis, with the estimate that the relative uncertainty of the measured thermal conductivity increases above 480 K by the additive amount of 1.0 % due to extrapolation in the wire calibration.

## 3. Results

**Fig. 4:**
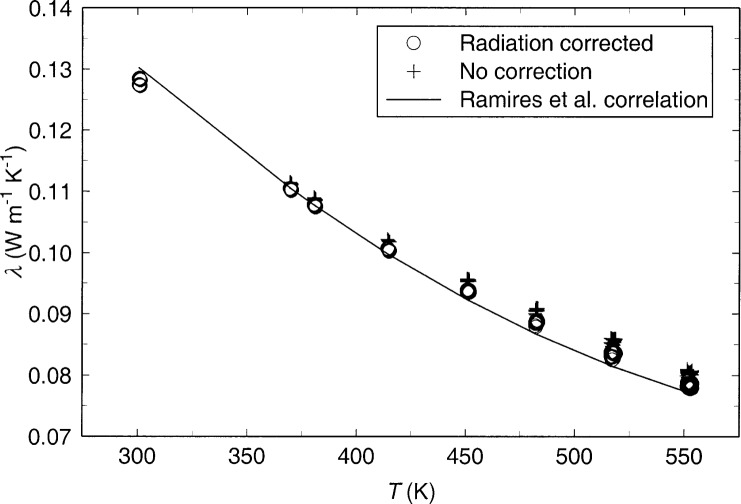
Thermal conductivity of saturated liquid toluene as a function of temperature. The correction for thermal radiation increases with *T*^3^ to about 3 % at 550 K.

The thermal conductivity results for the purified toluene sample are given in Table 2 and are shown in Fig. 4. The results in Table 2 have been corrected for thermal radiation, as were our previous results using bare platinum hot wires [4], and with the same empirical optical parameters that were found for the fluid using the previous platinum hot wires [5]. Since the power divided by length was not equal for the long and short wires after the isotherm at 550 K, as shown in Fig. 2, the data from the decreasing temperature portion of the temperature cycle are not reported. There are 184 thermal conductivity data points at temperatures from 300 K to 550 K.

### 3.1 Repeatability at High Temperatures

**Fig. 5:**
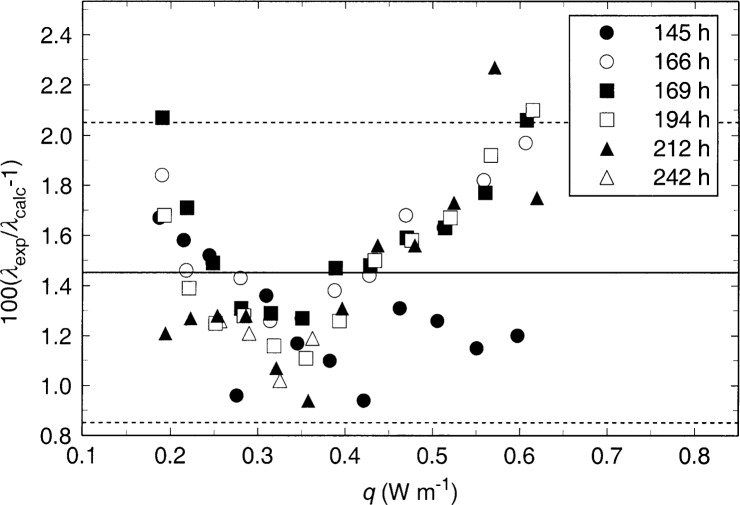
Thermal conductivity of saturated liquid toluene as a function of applied power near 550 K. Different plot symbols designate the average elapsed time of the experiment series. The mean deviation of the data (solid line) is 1.45 % higher than the earlier reference standard, and the scatter (dashed lines) is ±0.6 % at the level of 95 % confidence.

The isotherm at 550 K is quite interesting since it includes several replications, with a wide range of power levels, over the course of four days. During this time, the resistance of the long hot wire increased by 5.6 Ω and that of the short hot wire increased by 1.9 Ω. In addition, since this increase in resistance was not proportional to the wire lengths, the power generation of the two wires differed by up to 1.6 %. Dispite these complications, there seems to be little additional scatter in the thermal conductivity results measured during this 4 day period. In Fig. 5, deviations between the results for the 550 K isotherm are plotted relative to the reference standard of Ramires et al. [2] as a function of applied power level at 550 K. The mean deviation of the data (solid line) is 1.45 % higher than the earlier reference standard, and the scatter (dashed lines) is ±0.6 % at the level of 95 % confidence. No trend is noted with respect to time throughout this four-day period. Convection in the sample occurs at shorter times for experiments with higher powers and correspondingly larger temperature rises. Consistency between thermal conductivity results at different power levels is considered a good indication that there was no significant convection during the measurements and that compensation for the wire’s end effects was achieved. Data at power levels above 0.5 Wm^−1^ appear to have some influence due to the onset of convection since this isotherm is relatively close to the critical point of toluene at 593.95 K. The contribution of convection on the apparent thermal conductivity appears to be less than 0.5 % at even the highest power levels.

### 3.2 Uncertainty Assessment

The contribution of thermal radiation to measurements of the thermal conductivity of fluids such as toluene has been a topic of debate for many years. An empirical technique has been described for correcting for thermal radiation in transient hot-wire measurements [5]. Empirical optical parameters have been reported for toluene [5] at these same conditions based on measurements with platinum hot wires 12.7 μm in diameter. If this radiation correction is valid, then these same optical parameters should apply to the present case of a tantalum wire 25 μm in diameter. The radiation correction assumes that thermal emission from the wire is small compared to emission from the expanding thermal front in the fluid, so there should be little effect due to changing the emissivity of the wire material. The contribution of thermal radiation is insignificant at 300 K, increases as *T*^3^ and is estimated to be 3 % at 550 K. The experimental thermal conductivity data with and without this radiative correction are shown in Fig. 4.

**Fig. 6:**
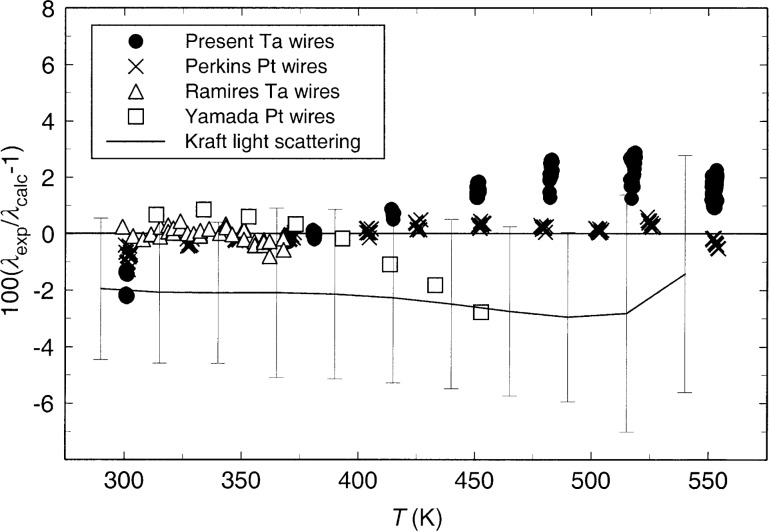
Deviations between the thermal conductivity data, [4,6,8,18] and present work, and the reference standard of Ramires et al. [2].

Deviations between the reference standard of Ramires et al. [2] and the present data are shown in Fig. 6. High-temperature data sets from light scattering [6] as well as transient hot-wire experiments using bare platinum [4,18] and anodized tantalum[8] hot wires are also compared in this figure. The data of Kraft et al. [6] were obtained from thermal diffusivities by light scattering using the equation of state of Goodwin [7]. These light-scattering data are free from uncertainty due to thermal radiation but are believed to have a relative uncertainty of 2.5 % in thermal diffusivity. The uncertainty in the thermal conductivity obtained from the expression λ = *aρC_p_* is estimated to vary between 3 % and 4.2 % since the uncertainties in ρ and *C_p_* from the equation of state [7] are 0.2 % for ρ and varies from 0.2 % to 3 % for *C_p_*. The thermal conductivity obtained from light scattering is offset from the direct thermal conductivity measurements by about 2 % to 3 %, but agreement is still within the combined uncertainty of the data sets. It is unlikely that the offset is due to thermal radiation since it is nearly constant and thermal-radiative errors should increase as *T*^3^.

No systematic difference between transient hot-wire measurements using bare and anodized tantalum hot wires is apparent in Fig. 6. The present tantalum hot-wire data are lower than the other transient data [4,8,18] at 300 K by about 2 %. This is partly due to larger cell fluctuations of temperature in the furnace containing the hot-wire cell near ambient temperature. The remainder of this difference is likely due to the drift in calibration of the tantalum hot wires at elevated temperatures, as shown in Fig. 3. The uncertainty of the present tantalum measurements are evaluated as 1 % at 300 K, 0.5 % from 369 K to 480 K, and 1.5 % from 480 K to 550 K. The transient hot-wire data using platinum or anodized tantalum wires agree within their combined uncertainties over the entire temperature range, with the exception of the data of Yamada et al. [18] at temperatures above 400 K. The purity of the toluene sample used in this case [18] was stated to be 99.7 % by the supplier. The purity of the toluene sample used in the light scattering study [6] was stated to be 99.9 % by the supplier. The transient hot-wire data of Perkins et al. [4] and Ramires et al. [8] were made on purified samples of toluene as described in the present work.

## 4. Conclusions

The present measurements demonstrate that anodized-tantalum hot wires can be used to make absolute measurements of the thermal conductivity a liquid from 300 K to 550 K. Previous studies with anodized-tantalum hot wires have been limited to temperatures below 370 K [8]. The present transient hot-wire measurements using anodized-tantalum hot wires have a larger uncertainty in the temperature extremes than our previous measurements using bare tantalum hot wires [4] over the same temperature range. This is primarily due to drift in the resistance calibration of the anodized tantalum hot wires at high temperatures. Use of tantalum lead wires may reduce or eliminate this problem in the future and allow accurate measurements of the fluid thermal diffusivity. It was also noted during the experiments that convection occurs earlier, and at lower power levels, as the wire diameter increases. Thus, experiments must be done with lower levels of applied power (smaller temperature rises) when the larger tantalum hot wires are used.

The big advantage of anodized-tantalum hot wires is for measurement of electrically conducting fluids, and this is not a problem in the case of toluene. The anodized tantalum hot wires have a geometry and emissivity different from those of platinum, so the present measurements support the validity of the thermal-radiation correction for absorbing fluids [5]. There is no significant temperature trend in deviations between the present radiation-corrected thermal-conductivity data and thermal-diffusivity data derived from light-scattering measurements of thermal diffusivity. This again supports the validity of the radiation correction [5], since the contribution of thermal radiation is expected to increase with *T*^3^.

## Figures and Tables

**Table 1:** Calibration coefficients for the anodized tantalum hot wires

Temp./Wire	*A*_1_ (Ω)	*A*_2_ (Ω K^−1^)	*A*_3_ (Ω K^−2^)
450 K / Long	6.425 77	0.179 655	−0.595 598 × 10^−5^
450 K / Short	1.811 64	0.463 252 × 10^−1^	−0.102 980 × 10^−5^
480 K / Long	6.642 31	0.178 426	−0.424 702 × 10^−5^
480 K / Short	1.902 16	0.458 112 × 10^−1^	−0.315 381 × 10^−6^
515 K / Long	9.252 54	0.164 042	0.150 564 × 10^−4^
515 K / Short	2.666 52	0.415 993 × 10^−1^	0.533 726 × 10^−5^
550 K / Long	9.471 42	0.162 877	0.165 577 × 10^−4^
550 K / Short	2.697 78	0.414 329 × 10^−1^	0.555 170 × 10^−5^

**Table 2:** Thermal conductivity of toluene near the saturated liquid line

*T* (K)	*p* (MPa)	λ (W m^−1^ K^−1^)	*q* (W m^−1^)	*T* (K)	*p* (MPa)	λ (W m^−1^ K^−1^)	*q* (W m^−1^)
301.248	3.246	0.128 18	0.344 40	381.226	0.092	0.107 47	0.391 08
301.150	3.252	0.128 20	0.317 30	381.078	0.088	0.107 42	0.359 11
301.051	3.253	0.128 24	0.291 35	380.929	0.087	0.107 76	0.328 51
300.950	3.258	0.128 27	0.266 53	380.804	0.084	0.107 69	0.299 29
300.856	3.260	0.128 40	0.242 81	380.672	0.081	0.107 71	0.271 43
300.762	3.260	0.128 52	0.220 20	380.551	0.079	0.107 81	0.244 93
300.676	3.263	0.128 49	0.198 70	380.429	0.078	0.107 73	0.219 80
300.595	3.266	0.128 45	0.178 31	381.304	0.293	0.107 64	0.424 55
301.231	0.077	0.127 25	0.344 42	381.166	0.292	0.107 50	0.391 11
301.134	0.075	0.127 23	0.317 30	381.024	0.290	0.107 53	0.359 06
301.030	0.067	0.127 35	0.291 32	380.889	0.290	0.107 76	0.328 46
300.934	0.059	0.127 28	0.266 49	380.759	0.288	0.107 77	0.299 25
300.834	0.053	0.127 36	0.242 78	380.620	0.288	0.107 85	0.271 38
300.745	0.047	0.127 35	0.220 17	380.509	0.286	0.107 81	0.244 89
300.661	0.044	0.127 49	0.198 68	380.391	0.285	0.107 94	0.219 76
300.580	0.040	0.127 44	0.178 29	414.154	0.272	0.100 76	0.224 35
370.214	0.101	0.110 17	0.413 57	414.288	0.271	0.100 71	0.250 66
370.078	0.101	0.110 14	0.381 02	414.416	0.271	0.100 64	0.278 46
369.941	0.101	0.110 13	0.349 85	414.559	0.270	0.100 60	0.307 76
369.820	0.101	0.110 33	0.320 03	414.709	0.270	0.100 43	0.338 56
369.698	0.101	0.110 38	0.291 55	414.858	0.269	0.100 23	0.370 81
369.578	0.101	0.110 48	0.264 41	415.036	0.269	0.100 38	0.404 56
369.467	0.101	0.110 49	0.238 59	415.201	0.269	0.100 37	0.439 75
369.368	0.101	0.110 51	0.214 11	451.768	0.522	0.093 51	0.494 27
370.250	0.324	0.110 24	0.413 58	451.575	0.522	0.093 69	0.455 48
370.115	0.310	0.110 21	0.381 03	451.393	0.522	0.093 73	0.418 30
369.982	0.297	0.110 11	0.349 82	451.223	0.522	0.093 81	0.382 68
369.859	0.286	0.110 39	0.320 01	451.044	0.522	0.093 77	0.348 64
369.739	0.276	0.110 39	0.291 52	450.874	0.522	0.093 76	0.316 18
369.611	0.267	0.110 43	0.264 38	450.720	0.521	0.093 71	0.285 32
369.500	0.259	0.110 43	0.238 57	450.573	0.521	0.093 68	0.256 05
369.393	0.252	0.110 50	0.214 09	451.732	0.859	0.093 87	0.494 47
381.373	0.093	0.107 52	0.424 40	451.539	0.858	0.093 74	0.455 50
451.364	0.858	0.093 90	0.418 24	552.555	2.567	0.077 98	0.382 26
451.172	0.857	0.093 45	0.382 64	552.367	2.567	0.078 05	0.344 96
451.017	0.857	0.093 60	0.348 60	552.182	2.566	0.078 22	0.309 59
450.855	0.854	0.093 90	0.316 16	552.004	2.565	0.077 92	0.276 13
450.711	0.855	0.093 91	0.285 31	551.852	2.563	0.078 39	0.244 58
450.566	0.855	0.093 93	0.256 03	551.688	2.562	0.078 45	0.214 95
451.752	0.802	0.093 62	0.494 80	551.547	2.562	0.078 54	0.187 25
451.567	0.802	0.093 91	0.455 83	553.467	2.628	0.078 57	0.606 40
451.382	0.802	0.093 87	0.418 52	553.200	2.632	0.078 48	0.558 67
451.213	0.803	0.093 79	0.382 85	552.981	2.635	0.078 35	0.512 97
451.037	0.804	0.093 72	0.348 77	552.771	2.637	0.078 42	0.469 24
450.876	0.803	0.094 02	0.316 29	552.550	2.640	0.078 25	0.427 50
450.711	0.804	0.093 53	0.285 41	552.365	2.641	0.078 22	0.387 73
450.566	0.805	0.093 72	0.256 14	552.165	2.643	0.078 15	0.349 90
483.230	0.922	0.088 56	0.525 51	551.991	2.645	0.078 16	0.314 01
483.013	0.917	0.088 51	0.484 20	551.816	2.646	0.078 32	0.280 08
482.805	0.913	0.088 46	0.444 67	551.656	2.647	0.078 39	0.248 07
482.598	0.910	0.088 76	0.406 78	551.501	2.648	0.078 38	0.218 03
482.413	0.908	0.088 45	0.370 60	551.367	2.649	0.078 70	0.189 92
482.234	0.906	0.087 86	0.336 12	553.540	2.657	0.078 63	0.607 76
482.064	0.905	0.088 64	0.303 31	553.293	2.658	0.078 43	0.559 88
481.906	0.903	0.088 08	0.272 20	553.057	2.658	0.078 34	0.514 02
483.220	1.574	0.088 83	0.525 69	552.830	2.659	0.078 33	0.470 20
483.019	1.574	0.088 93	0.484 21	552.604	2.659	0.078 27	0.428 39
482.808	1.574	0.088 62	0.444 55	552.393	2.659	0.078 29	0.388 53
482.614	1.572	0.088 99	0.406 68	552.193	2.659	0.078 15	0.350 62
482.433	1.572	0.088 58	0.370 53	552.001	2.659	0.078 19	0.314 68
482.263	1.571	0.088 94	0.336 08	551.814	2.659	0.078 22	0.280 69
482.086	1.571	0.088 52	0.303 29	551.652	2.658	0.078 39	0.248 62
481.934	1.572	0.088 45	0.272 17	551.492	2.659	0.078 58	0.218 50
518.628	1.534	0.083 52	0.562 32	551.342	2.659	0.078 88	0.190 33
518.393	1.533	0.083 38	0.518 02	553.646	2.635	0.078 65	0.614 86
518.162	1.533	0.083 24	0.475 68	553.388	2.635	0.078 54	0.566 49
518.032	1.533	0.083 08	0.435 19	553.139	2.635	0.078 37	0.520 16
517.748	1.533	0.082 75	0.396 48	552.896	2.634	0.078 32	0.475 86
517.546	1.532	0.083 05	0.359 60	552.668	2.635	0.078 28	0.433 54
517.348	1.532	0.082 93	0.324 50	552.445	2.634	0.078 12	0.393 21
517.179	1.532	0.082 96	0.291 20	552.240	2.634	0.078 02	0.354 85
517.010	1.532	0.082 51	0.259 72	552.047	2.634	0.078 08	0.318 45
516.792	1.532	0.083 03	0.230 05	551.852	2.634	0.078 20	0.284 03
516.638	1.532	0.082 92	0.202 18	551.684	2.634	0.078 19	0.251 58
516.501	1.532	0.083 14	0.176 11	551.517	2.634	0.078 32	0.221 11
518.578	2.143	0.083 67	0.562 45	551.367	2.633	0.078 57	0.192 61
518.347	2.144	0.083 66	0.518 04	553.163	2.619	0.078 43	0.619 33
518.147	2.143	0.083 57	0.475 63	553.346	2.619	0.078 82	0.571 03
517.928	2.142	0.083 73	0.435 12	553.083	2.620	0.078 42	0.524 29
517.670	2.142	0.083 64	0.396 47	552.840	2.620	0.078 31	0.479 62
517.482	2.141	0.083 50	0.359 57	552.612	2.620	0.078 34	0.436 95
517.275	2.142	0.083 63	0.324 48	552.388	2.620	0.078 17	0.396 28
517.094	2.142	0.083 67	0.291 19	552.169	2.621	0.077 89	0.357 63
516.915	2.142	0.083 74	0.259 73	551.974	2.621	0.078 02	0.320 95
516.764	2.143	0.083 71	0.230 05	551.784	2.621	0.078 20	0.286 26
516.606	2.143	0.083 74	0.202 18	551.607	2.621	0.078 22	0.253 55
516.467	2.143	0.083 80	0.176 10	551.436	2.622	0.078 24	0.222 84
553.594	2.565	0.077 94	0.597 49	551.285	2.622	0.078 20	0.194 11
553.379	2.567	0.077 93	0.550 59	552.155	2.642	0.078 09	0.362 33
553.178	2.566	0.078 04	0.505 64	551.949	2.642	0.077 98	0.325 12
552.967	2.567	0.078 10	0.462 60	551.762	2.642	0.078 15	0.289 94
552.740	2.567	0.077 83	0.421 46	551.579	2.642	0.078 21	0.256 80
